# Patient communication (SMS) in ICU

**DOI:** 10.4103/0972-5229.60178

**Published:** 2009

**Authors:** P. S. R. K. Haranath

**Affiliations:** Retired Director of Medical Education, Andhra Pradesh, India

Dear Editor,

Once a patient is admitted into ICU, and relatives blocked at the entrance, the entire care is in control of the ICU staff. An unconscious patient is put on a ventilator, leads established for recording ECG, temperature, respiration etc and infusion systems and other equipment used for the management of these very sick patients. The attention of the staff then mostly is on the monitoring systems. However, the problems of a conscious patient on a ventilator are different. While the monitoring systems reflect the functioning of the patient's biological system, they do not indicate information on other sensations/ feelings of the patient like feeling cold, pain etc. Also a patient's desire to communicate with the staff with speech is not possible because of the endotracheal tube. This is complicated by the fact that the attention of the staff on entering an ICU cubicle, is first directed to the monitoring panels and finally to the intubated, anxious and conscious patient. Often the patient wishes to communicate something, a doubt, a question or request. Some patients can be made to write with difficulty on a piece of paper, sometimes not coherent and not possible most often.

I expressed some of these problems faced by patients in a previous letter to the editor.[1]

I have the following suggestions for consideration:

- Develop a device like an ‘online key board and screen’ to be held in front of the patient who can by himself or prompted by guidance to express an ‘SMS’ for action. The manufacturers of such sophisticated instruments in the ICU can easily design one such valuable aid to be used by the staff and patient. It gives feedback of information not seen with the monitors and an opportunity to the patient to communicate his/her wishes which may sometimes be their last.
- Develop alternate procedures to measure arterial blood gas values to help early removal of the tracheal tube. Frequent femoral arterial punctures are distressing. Some steps are being made in this direction with ear lobe samples.[2,3] Alternate parameters, tests, measurements or criteria can be explored to take the patient off the mechanical ventilator at the earliest.

ICUs are rapidly developing new fields of patient care. Patient communication is a vital area of concern as complaints often emanate from the patient and the relations.
